# Vapor Pressure Formulation for Water in Range 0 to 100 °C. A Revision

**DOI:** 10.6028/jres.080A.071

**Published:** 1976-10-01

**Authors:** Arnold Wexler

**Affiliations:** Institute for Basic Standards, National Bureau of Standards, Washington, D.C. 20234

**Keywords:** Clapeyron equation, saturation vapor pressure over water, steam, vapor pressure, vapor pressure of water, virial coefficients

## Abstract

In 1971 Wexler and Greenspan published a formulation for the vapor pressure of water encompassing the temperature range 0 to 100 °C. In this paper a revision is made of that earlier formulation to make it consistent with the definitive experimental value of the vapor pressure of water at its triple point recently obtained by Guildner, Johnson, and Jones. The two formulations are essentially identical at temperatures from 25 to 100 °C.

For temperatures below 25 °C the new formulation predicts values that are higher than the 1971 formulation. At the triple point, the vapor pressure given by the new formulation is 611.657 Pa whereas the value given by the 1971 formulation is 611.196 Pa. A table is given of the vapor pressure as a function of temperature at 0.1-deg intervals over the range 0 to 100 °C on the International Practical Temperature Scale of 1968, together with values of the temperature derivative at 1-deg intervals.

## 1. Introduction

In 1971, Wexler and Greenspan [1]^1^ derived an equation for computing the vapor pressure of water over the temperature range 0 to 100 °C. They integrated the Clapeyron equation, using the accurate calorimetric data of Osborne, Stimson and Ginnings [2], and the Goff and Gratch formulation [3] for the virial coefficients of water vapor, to obtain a smoothing function that has a rational basis. Calculated values of vapor pressure agreed with the very precise measurements of Stimson [4] to within 7 ppm from 25 to 100 °C. Comparable measurements below 25 °C were not available for comparison. Recently, Guildner, Johnson, and Jones [5] completed a series of highly accurate measurements of the vapor pressure of water at its triple point. They obtained the definitive value of 611.657 Pa^2^ with an estimated total uncertainty at 99 percent confidence limits (3 sigmas plus the estimated systematic errors) of ±0.010 Pa (±16 ppm). The 1971 equation predicts a vapor pressure at the triple point of 611.196 Pa, a value which is lower by 0.461 Pa (754 ppm). The 1971 formulation, therefore, was reexamined and revised so that it is now consistent with this new experimental triple point value as well as with the older Stimson measurements. By using new gas thermometry data [6] it was possible to derive an equation for vapor pressure as a function of thermodynamic temperature and also of the International Practical Temperature Scale of 1968.

## 2. Derivation

A modified version of the Clapeyron equation [7] is chosen as the starting point:

$$\frac{dp}{dT}=\frac{\gamma }{Tv}$$ (1)

where *p* is the pressure of the saturated vapor, *v* is the specific volume of the saturated vapor, *T* is the absolute thermodynamic temperature, *T* is an experimentally measured calorimetric quantity not substantially different from the latent heat of vaporization [7], and *dp/dT* is the derivative of the vapor pressure with respect to the absolute temperature. The specific volume, obtained from the virial equation of state for water vapor, is

$$v=\frac{RTZ}{p}=\frac{RT}{p}\left(1+B^{\prime }p+C^{\prime }p^{2}+\ldots \right)$$ (2)

where *R* is the gas constant for water vapor, *Z* is the compressibility factor, *B*′ is the second pressure-series virial coefficient and *C*′ is the third pressure-series virial coefficient. When eq (2) is substituted into eq (1) it follows that

$$\frac{dp}{p}=\frac{\gamma }{RT^{2}Z}dT$$ (3)

After performing several simple mathematical manipulations and integrating, eq (3) becomes

$$\int_{p_{0}}^{p}d\left(\ln p\right)=\int_{T_{0}}^{T}\frac{\gamma }{RT^{2}}dT-\int_{T_{0}}^{T}\frac{\gamma }{RT^{2}}\left(\frac{Z-1}{Z}\right)dT$$ (4)

where *p*_0_ and *p* are the initial and final vapor pressures corresponding to temperatures *T*_0_ and *T*, respectively.

The quantity *γ* is represented by the polynomial eauation

$$\gamma =a_{0}+a_{1}T+a_{2}T^{2}+a_{3}T^{3}$$ (5)

where *a*_0_, *a*_1_, *a*_2_ and *a*_3_ are constants. Inserting eq (5) into the first integral on the right-hand side of eq (4) yields

$$\overset{p}{\underset{p_{0}}{\int }}d\left(\ln p\right)=-\frac{a_{0}}{R}\left(\frac{1}{T}-\frac{1}{T_{0}}\right)+\frac{a_{1}}{R}\ln\frac{T}{T_{0}}+\frac{a_{2}}{R}\left(T-T_{0}\right)+\frac{a_{3}}{2R}\left(T^{2}-T_{0}{\;}^{2}\right)-\overset{T}{\underset{T_{0}}{\int }}\frac{\gamma }{RT^{2}}\left(\frac{Z-1}{Z}\right)dT$$ (6)

The constants in eq (5) were obtained by fitting the Osborne, Stimson, and Ginnings weighted mean values of *γ* [2] from 0 to 150 °C by the method of least squares after converting the reported temperatures, given on the International Temperature Scale of 1927 (ITS–27), to absolute thermodynamic temperatures and the reported heat units of international joules to (absolute) joules.^3^ The conversion of temperatures on ITS–27 to thermodynamic temperatures will be reserved for later discussion. The coefficients of eq (5) have the following values: *a*_0_=3423.8440, *a*_1_ = −5.2277204, *a*_2_ = 0.9855719×10^−2^, and *a*_3_ = −.11305118×10^−4^.

The triple point vapor pressure *p*_0_=611.657 Pa and the absolute temperature at the triple point *T*_0_=273.16 K [5] were selected as lower limits of integration for substitution into eq (6). The gas constant for water vapor, *R*, is 0.461520 joules per gram kelvin and was derived from the CODATA recommended value [8] of 8.31441 joules per mole kelvin for the univeral gas constant and 18.01528 grams for the molecular weight of naturally occurring water on the unified carbon-12 scale.^4^

The Keys equation [12] was used for the second pressure-series virial coefficient. When converted to SI units, compatible with eq (2), it takes the form

$$B^{\prime }=\left[\frac{0.44687}{T}-\left(\frac{565.965}{T^{2}}\right)10^{\left(\frac{100800}{34900+T^{2}}\right)}\right]\times 10^{-5}$$ (7)

where *B*′ is in units of reciprocal pressure, (Pa)^−1^. From the experimental vapor pressure data of Stimson [4] and of Guildner, Johnson, and Jones [5], and the calorimetric data of Osborne, Stimson, and Ginnings [2], the saturated specific volumes of water vapor were calculated using eq (1). These volumes, when inserted into eq (2), together with *B*′ from eq (7), yielded values of an effective third pressure-series virial coefficient *C′*. These computed values of *C*′ were fitted by the method of least squares to the equation

$$-\ln C^{\prime }=\left(d_{0}+\frac{d_{1}}{T}+\frac{d_{2}}{T^{2}}+\frac{d_{3}}{T^{3}}\right)$$ (8)

yielding *d*_0_ = 0.311018×10^3^, *d*_1_= −0.349634×10, *d*_2_=0.116994×10^9^, and *d*_3_= −0.126779×10^11^. Equation (8) is valid only from the triple point to the steam point and is expressed in SI units of the square of the reciprocal pressure, (Pa)^−2^, compatible with eq (2).

Because eq (6) is an implicit function in *p* calculations of *p* were made by iteration. The integral on the right was evaluated numerically at 20 mK intervals by means of the trapezoidal rule [13]. Iteration at each interval was terminated when successive values of *p* differed by less than 0.1 ppm. Fifty-one numerical values of the right-hand integral of eq (6), at 2-kelvin intervals starting at the triple point, were fitted by the method of least squares to the equation

$$\int_{T_{0}}^{T}\frac{\gamma }{RT^{2}}\left(\frac{Z-1}{Z}\right)dT=b_{0}\left(\frac{1}{T}-\frac{1}{T_{0}}\right)+b_{2}\left(T-T_{0}\right)+b_{3}\left(T^{2}-T_{0}^{2}\right)+b_{1}\ln\frac{T}{T_{0}}$$ (9)

yielding *b*_0_=−0.13750137×10^4^,

*b*_1_=−0.14185668×10^2^,

*b*_2_=0.49593509×10^−1^, and *b*_3_=−0.29488830×10^−4^. It should be noted that the *b*’s are replacements for the parameters of equations (7) and (8). By combining terms on the right-hand side of eq (6), and then integrating the lefthand side, an explicit equation was obtained, namely,

$$\begin{array}{l} \ln p=-\left(\frac{a_{0}}{R}+b_{0}\right)\left(\frac{1}{T}-\frac{1}{T_{0}}\right)+\left(\frac{a_{2}}{R}-b_{2}\right)\left(T-T_{0}\right)\\ +\left(\frac{a_{3}}{2R}-b_{3}\right)\left(T^{2}-T_{0}^{2}\right)+\left(\frac{a_{0}}{R}-b_{1}\right)\ln\frac{T}{T_{0}}+\ln p_{0} \end{array}$$ (10)

which, with the appropriate constants, reduces to

$$\ln p=\sum_{i=0}^{3}c_{i}T^{t-1}+c_{4}\ln T$$ (11)

where *c*_0_=−0.60436117×10^4^, *c*_1_=0.189318833×10^2^, *c*_2_=−0.28238594×10^−1^, *c*_3_=0.17241129×10^−4^, and *c*_4_=0.2858487×10^1^.

At the steam point, the value of the vapor pressure given by eq (11) is greater than the defined value of 101325 Pa by 3.4 Pa (38 ppm). By introducing an arbitrary but minor change in the coefficients *c*_1_, *c*_2_, and *c*_3_, the equation was adjusted to pass through 101325 Pa with negligible effect on the intermediate vapor pressures. The three adjusted coefficients now have the following values: *c*_1_ = 0.1893292601×10^2^, *c*_2_= −0.28244925×10^−1^, and *c*_3_=0.17250331×10^−4^.

## 3. Conversion to IPTS–68

Over the range 0 to 100 °C, the temperature in degrees Celsius has the same numerical value on the International Temperature Scale of 1972, (ITS–27), the International Temperature Scale of 1948 (ITS–48), and the International Practical Temperature Scale of 1948 (IPTS–48). On the other hand, over the same range, the temperature on the International Practical Temperature Scale of 1968 (IPTS–68) differs from that of ITS–27, ITS–48 and IPTS–48. Using the corrections given by Riddle, Furukawa and Plumb [14], temperatures on these latter three scales were converted to IPTS–68.

Guildner and Edsinger [6] have made a series of measurements on the realization of the thermodynamic temperature scale (TTS) from 273.2 to 730.44 K by means of gas thermometry. They fitted their data to an equation of the form

$$T_{68}-T=\sum_{i=0}^{4}\alpha_{i}T^{i-2}$$ (12)

where *T*_68_ is the absolute temperature in kelvins on IPTS–68. They obtained the following values for the coefficients: *α*_0_=0.1192951052×10^6^, *α*_1_=−0.119917011×10^4^, *α*_2_=0.427014907×10^1^, *α*_3_=−0.637942023×10^−2^, and *α*_4_=0.353749196×10^−5^. The residual standard deviation of the fit was 1.57 mK.

Their data were refitted up to 472.78 K to an equation of the form

$$t_{68}-t=\sum_{i=0}^{3}\beta_{i}{\left(t-0.01\right)}^{t}$$ (13)

which imposed the constraint that *t*_68_=*t* at the triple point and where *t*_68_ and *t* are the temperatures in degrees Celsius on IPTS–68 and TTS, respectively. This equation was then converted to absolute temperatures, yielding

$$T_{68}-T=\sum_{i=0}^{3}\rho_{i}T^{i}$$ (14)

where *ρ*_0_=0.4949479, *ρ*_1_=−0.46352557×10^−2^, *ρ*_2_=0.13852156×10^−4^ and *ρ*_3_=−0.12872954×10^−7^. Over the range from 273.15 to 373.16 K (the range of interest here), the temperatures calculated by eq (14) do not differ from those calculated by eq (12) by more than 0.79 mK; the standard deviation of the difference between *T*_68_ as calculated by eq (14) and *T*_68_ as measured by Guildner and Edsinger is 1.5 mK.

In the range from the triple point to the steam point, the numerical values on IPTS–68 become progressively larger than those on TTS at identical temperatures. At the steam point *T*_68_ is greater than *T* by about 25 mK (~67 ppm).

One way of calculating the vapor pressure is to convert IPTS–68 to TTS temperatures via eq (14) and then to insert these computed thermodynamic temperatures into eq (11). Alternatively, eq (11) can be transformed to IPTS–68 by substituting eq (14) into eq (11). This algebraic manipulation yields

$$\ln p=\sum_{i=0}^{6}g_{i}T_{68}^{i-2}+g_{7}\ln T_{68}$$ (15)

The coefficients are given in table 1.

This algebraic conversion increased the number of terms from five in eq (11) to eight in eq (15). The feasibility of simplifying eq (15) was investigated. The procedure adopted was to fit by the method of least squares, 102 values of vapor pressure, generated by eq (15) at one-kelvin intervals starting at the ice point, to an equation of the form

$$\ln p=\sum_{i=1}^{n}g_{1}T_{68}^{i-2}+g_{n+1}\ln T_{68}$$ (16)

for 3⋜*n*⋜6 with and without the ln *T*_68_ term. Equation (16) is analogous to eq (15) except for the number of terms. For *n*=4 and including the ln *T*_68_ term, eq (16) yields values of vapor pressure which differ from those calculated using eq (15) by 0.4 ppm or less. For *n*=4 but without the ln *T*_68_ term, eq (16) yields values which differ from those calculated using eq (15) by 20 ppm or less. For convenience these two versions of eq (16) will be designated eq (16a) and eq (16b) respectively. The coefficients are given in table 1. The use of more terms does not improve the agreement whereas decreasing *n* to 3 degrades the agreement by an order of magnitude or more.

## 4. Results

Because of the high precision and internal consistency of the Stimson measurements the adequacy of the 1971 formulation was judged primarily on its agreement with the Stimson data. The same Stimson data, augmented by the definitive vapor pressure measurement at the triple point of Guildner, Johnson, and Jones, will be used to judge the present formulation. Differences in vapor pressure between eq (15) and these two sets of measurements are given in table 2, together with the experimental uncertainty of the measured values. Equation (15) obviously yields the Guildner, Johnson, and Jones value of the vapor pressure at the triple point because it was constrained to pass through this value. It also yields vapor pressures which are in agreement with Stimson’s values to within one standard deviation of the latter except at 70 °C where the agreement is within two standard deviations. The maximum difference, 43 ppm, occurs at 25 °C.

A comparison between this formulation, using eq (15) as a base line, and the 1971 formulation is shown in table 3. The two are in substantial agreement (⋜37 ppm) from 100 to 25 °C. Below 25 °C the difference between the two formulations increases from 37 ppm, reaching 754 ppm at the triple point.

Because the numerical values of temperatures on IPTS–68 are greater than those on TTS, it follows that the vapor pressures calculated on IPTS–68 with eq (15) are smaller than those calculated on TTS with eq (11) when using the same numerical values for temperature. As shown in table 4, for identical numerical values from 0 to 100 °C, eq (11) on TTS yields vapor pressures that increasingly exceed those calculated with eq (15) on IPTS–68 until, at 100 °C, the former is larger than the latter by 901 ppm. It is obvious that a substantial error will result unless the temperature is expressed on the appropriate scale for each equation.

**Figure 1:**
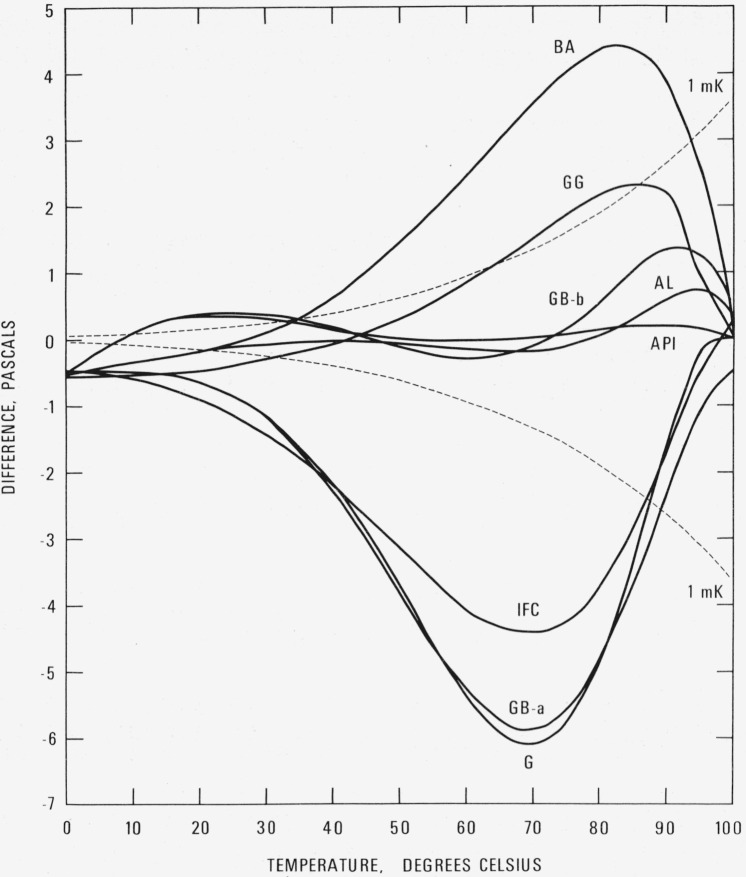
Comparison with other formulations. Vapor pressure difference [other-eq (15)] in pascals.
APIAmerican Petroleum Institute Research Project 44BABridgeman and AldrichGGerryGB-aGibson and Bruges, eq (a)GB-bGibson and Bruges, eq (b)GGGoff and GratchIFCInternational Formulation CommitteeALAmbrose and Lawrenson Dashed lines are difference curves for a temperature deviation of *±* 1 mK.

**Figure 2:**
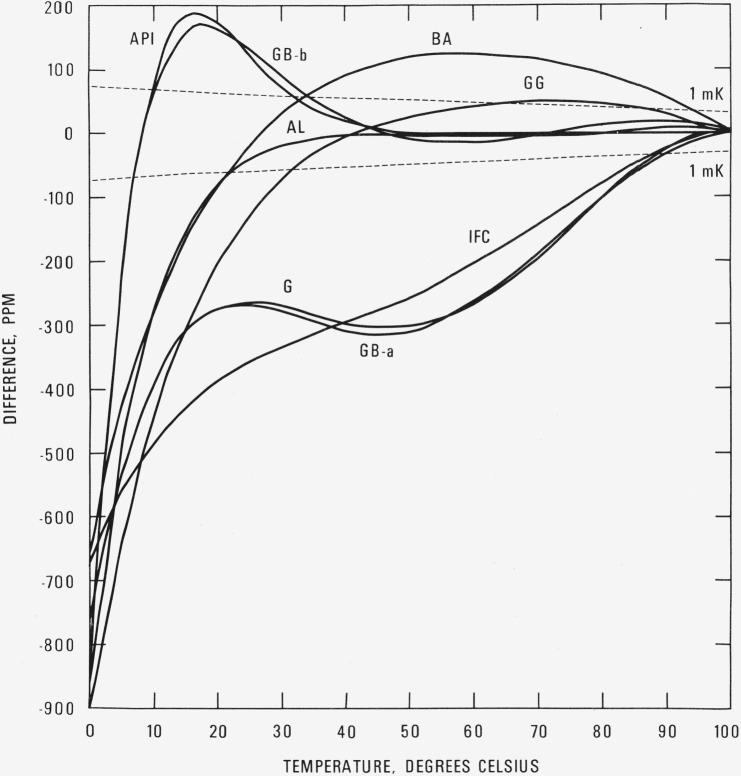
Comparison with other formulations. Vapor pressure difference $\left[\frac{\text{Other}-\text{eq}\;(15)}{\text{eq}\;(15)}\times 10^{6}\right]$ in ppm.
APIAmerican Petroleum Institute Research Project 44BABridgeman and AldrichGGerryGB-aGibson and Bruges, eq (a)GB-bGibson and Bruges, eq (b)GGGoff and GratchIFCInternational Formulation CommitteeALAmbrose and Lawrenson Dashed lines are difference curves for a temperature deviation of ± 1 mK.

A comparison of this formulation, using eq (15) as the base line, with several other formulations [15–20]^5^ in common use is shown in figures 1 and 2, with the differences given in pascals and parts per million, respectively. Appropriate temperature scale adjustments have been made to these formulations so that the calculated vapor pressures are on IPTS- 68. The important feature of this comparison is that as the temperature decreases below about 20 °C, these formulations predict values of vapor pressure that are consistently smaller than those obtained with eq (15). At the triple and ice points, the differences reach magnitudes of the order of 700 to 900 ppm.

In the earlier paper the 1971 formulation was compared with experimental data at and below the steam point. This will not be repeated except to note that, because this and the 1971 formulations yield essentially the same values of vapor pressure above 25 °C, the degree of accord with this formulation will be comparable.

There are two sets of modern vapor pressure measurements of water in the temperature range from 25 °C and below, those of Douslin [23] and Besley and Bottomley [24]. Differences between these data and eq (15) are given in tables 5 and 6. Douslin used an inclined dead weight piston page to make his measurements. He reported that his estimated systematic error varied from 0.31 Pa at −2.5 °C (609 ppm) to 0.81 Pa at 20.0 °C (346 ppm). His values are higher than those predicted by eq (15). Besley and Bottomley used a mercury manometer to make their series of measurements which they fitted to an empirical equation. They give no estimate of the overall systematic error of their measurements; rather they reported that the standard deviation of the fit was 1.7 mtorr (0.23 Pa) and used this as an estimate of their experimental imprecision. Their correlated value at the triple point is smaller in magnitude by about 906 ppm than that calculated with eq (15). Their values gradually approach those obtained with eq (15) until, at about 13.5 °C, the two agree. At 25 °C, the Besley and Bottomley values are higher by 57 ppm.

Using eq (15), vapor pressures in pascals were computed, as a function of temperature in degrees Celsius, on IPTS–68 from 0 to 100 °C. These computed values, as well as the derivative with respect to temperature, are given in table 7.

## 5. Discussion

In the 1971 paper, an analysis was presented of the uncertainties in such quantities and constants as *γ*, *R*, and *Z* and the contributions these uncertainties make in the calculation of *p.* A similar analysis will not be repeated here. Although in this work the computation was modified by using the triple point, rather than the steam point, as the lower limit of integration, substituting different virial coefficients for those of Goff and Gratch, and using the Guildner and Edsinger data for converting between TTS and IPTS–68, the conclusions of the earlier analysis are still valid: If the parameters entering into the computation are completely independent, then they must be known to an accuracy that is 1 to 2 orders of magnitude better than they are now known for thermodynamic calculations of vapor pressure to have an uncertainty comparable to the measurements of Stimson and of Guildner, Johnson and Jones. Equations (11) and (15) are presented, therefore, not as accurate theoretical representations of the properties of water but as smoothing functions that have a rational basis. Because of this rational basis, it is believed that the formulation has more validity for predicting (interpolating) vapor pressures at temperatures where corroborating experimental data of high accuracy do not exist than does one that is purely empirical.

Where vapor pressures are desired in terms of TTS, eq (11) should be used. Where vapor pressures are desired in terms of IPTS–68, then eq (15) or (16) should be used. We believe eq (15) is preferable because it has a thermodynamic basis whereas eqs (16a) and (16b) are empirical. However, eq (16b) without the ln *T*_68_ term has four coefficients and eq (16a) with the ln *T*_68_ term has five coefficients compared to the eight in eq (15). Therefore, where a reduction in coefficients is desirable, either version of eq (16) may be used. Both eqs (15) and (16) show comparable agreement with experimental data.

## Figures and Tables

**Table 1:** Coefficients to vapor pressure equations on IPTS–68

	Eq (15)	Eq (16a)	Eq (16b)
			
*g*_0_	−0.29912729×10^4^		
*g*_1_	−0.60170128×10^4^	−0.60951748×10^4^	−0.63536311×10^4^
*g*_2_	0.1887643854×10^2^	0.2116173595×10^2^	0.3404926034×10^2^
*g*_3_	−0.28354721×10^−1^	−0.27222404×10^−1^	−0.19509874×10^−1^
*g*_4_	0.17838301×10^−4^	0.16840790×10^−4^	0.12811805×10^−4^
*g*_5_	−0.84150417×10^−9^		
*g*_6_	0.44412543×10^−12^		
*g_n_*_+1_	0.2858487×10^1^	0.24505058×10^1^	

**Table 2:** Comparison of calculated vapor pressures with NBS measurements

Temperature	Vapor pressure		Difference		Experimental uncertainty^e^	
*t*_68_	*p*(cal)^a^	*p*(exp)	Δ*p*^d^	Δ*p*/*p*	1σ	3σ
						
°C	Pa	Pa	Pa	ppm	ppm	ppm
						
0.01	611.657	b611.657	0.00	0	±5. 3	±16
25	3168.74	c3168. 6	.14	43	44	132
40	7381.27	c7381.34	−.07	−9	20	60
50	12344.78	c12344.65	.13	10	15	45
60	19933.09	c19933. 05	.04	2	8	24
70	31177.32	c31177. 00	.32	10	7	21
80	47375.85	c47375. 2	.35	7	11	33
100	101324.99	c101325. 0	−.01	0	16	48

a Calculations made with eq (15).

b Guildner, Johnson, and Jones measurement.

c Stimson measurement.

d Δ*p*=*p*(calc) − *p*(exp).

e Uncertainty assigned by investigators to experimental values.

**Table 3:** Comparison with 1971 formulation

Temperature	Vapor pressure		Difference	
*t*_68_	*p*(1971)	*p*(1976)^a^	Δ*p*^b^	Δ*p*/*p*
				
°C	Pa	Pa	Pa	ppm
				
.00	610.752	611.212	0.460	754
.01	611.196	611.657	.461	754
5.00	872.045	872.469	.424	486
10.00	1227.57	1227.94	.37	300
15.00	1705.03	1705.32	.29	170
20.00	2338.34	2338.54	.20	86
25.00	3168.62	3168.74	.12	38
30.00	4245.15	4245.20	.05	12
35.00	5626.45	5626.45	.00	0
40.00	7381.29	7381.27	−.02	−3
45.00	9589.84	9589.84	.00	0
50.00	12344.73	12344.78	.05	4
55.00	15752.16	15752.26	.10	6
60.00	19932.93	19933.09	.16	8
65.00	25023.54	25023.74	.20	8
70.00	31177.15	31177.32	.17	5
75.00	38564.54	38564.59	.05	1
80.00	47374.98	47374.85	−.13	−3
85.00	57817.10	57816.73	−.37	−6
90.00	70119.59	70119.03	−.56	−8
95.00	84531.93	84531.40	−.53	−6
100.00	101324.97	101324.99	.02	0

a Computed with eq (15).

b *p*(1976)-*p*(1971).

**Table 4:** Comparison of vapor pressure calculated at identical numerical values on TTS and IPTS–68

Temperature	Vapor pressure		Difference	
*t*	*p*(T)^a^	*p*(T_68_)^b^	Δ*p*^c^	Δ*p*/*p*
				
°C	Pa	Pa	Pa	ppm
				
0	611.212	611.212	0.000	0
0.01	611.657	611.657	.000	0
5	872.487	872.469	.020	23
10	1228.01	1227.94	.07	57
15	1705.48	1705.32	.16	94
20	2338.87	2338.54	.33	141
25	3169.33	3168.74	.59	186
30	4246.21	4245.20	1.01	238
35	5628.09	5626.45	1.64	291
40	7383.83	7381.27	2.56	347
45	9593.69	9589.84	3.85	401
50	12350.41	12344.78	5.63	456
55	15760.30	15752.26	8.04	510
60	19944.32	19933.09	11.23	563
65	25039.10	25023.74	15.36	614
70	31197.99	31177.32	20.67	663
75	38591.97	38564.59	27.38	709
80	47410.57	57374.85	35.72	754
85	57862.72	57816.73	45.99	795
90	70177.50	70119.03	58.47	834
95	84604.88	84531.40	73.48	869
100	101416.33	101324.99	91.34	901

a Computed with eq (11) on TTS.

b Computed with eq (15) on IPTS–68.

c Δ*p=p*(*T*) − *p*(*T*_68_).

**Table 5:** Comparison with Douslin’s measurements

Series	Temperature *t*_68_	Vapor pressure		Difference		Experimental uncertainty^c^	
		*p*(D)	*p*(calc)^a^	Δ*p*^b^	Δ*p*/*p*	Δ*p*	Δ*p*/*p*
							
	°C	Pa	Pa	Pa	ppm	Pa	ppm
							
I	−2.50	508.8	508.7	0.1	200	±.31	±910
	0.00	611.4	611.2	.2	330	.32	520
	.01	612.1	611.6	.5	820	.33	540
	1.00	657.3	657.0	.3	460	.35	530
	2.00	706.1	705.9	.2	280	.35	500
	3.00	758.7	758.0	.7	920	.36	470
	4.00	813.7	813.5	.2	250	.37	450
	5.00	873.0	872.5	.5	570	.39	450
	7.50	1037.0	1036.8	.6	190	.43	420
	10.00	1229.1	1227.9	1.2	980	.45	370
	15.00	1706.6	1705.3	1.3	760	.57	330
II	7.50	1037.0	1036.8	.2	190	.43	410
	12.50	1448.5	1449.4	−.9	−620	.63	430
	17.50	2000.7	2000.1	.6	300	.73	360
	20.00	2339.5	2338.5	1.0	430	.81	350

a Computed with eq (15).

b *p*(D)-*p*(calc).

c Estimated maximum systematic errors assigned by investigator to experimental values.

**Table 6:** Comparison with Besley and Bottomley correlation

Temperature	Vapor pressure		Difference		Experimental uncertainty	
*t*_68_	*p*(*B*+*B*)	*p*(calc)^a^	Δ*p*^b^	Δ*p*/*p*	1*σ*	3*σ*
						
°C	Pa	Pa	Pa	ppm	ppm	ppm
						
0.00	610.659	611.212	−0.553	−907	±376	±1130
.01	611.103	611.657	−.554	−906	376	1130
1.00	656.555	657.069	−.514	−783	350	1051
2.00	705.476	705.949	−.473	−670	326	978
3.00	757.591	758.022	−.431	−569	304	911
4.00	813.079	813.467	−.388	−477	283	849
5.00	872.124	872.469	−.345	−395	264	791
6.00	934.920	935.222	−.302	−323	246	738
7.00	1001.67	1001.93	−.26	−259	230	689
8.00	1072.59	1072.80	−.21	−196	215	644
9.00	1147.89	1148.06	−.17	−148	200	600
10.00	1227.80	1227.94	−.14	−114	187	566
11.00	1312.58	1312.67	−.09	−69	175	526
12.00	1402.45	1402.51	−.06	−43	164	461
13.00	1497.69	1497.72	−.03	−20	154	492
14.00	1598.57	1598.56	.01	6	144	432
15.00	1705.36	1705.32	.04	23	135	405
16.00	1818.36	1818.29	.07	38	126	379
17.00	1937.87	1937.78	.09	46	119	356
18. 00	2064.20	2064.09	.11	53	111	334
19.00	2197.70	2197.57	.13	59	105	314
20.00	2338.69	2338.54	.15	64	98	295
21.00	2487.53	2487.37	.16	64	92	277
22.00	2644.59	2644.42	.17	64	87	261
23.00	2810.24	2810.06	.18	64	82	246
24.00	2984.88	2984.70	.18	60	77	231
25.00	3168.92	3168.74	.18	57	73	218

a Computed with eq (15).

b *p* (*B*+*B*) – *p*(calc).

**Table 7:** Saturation vapor pressure over water (IPTS—68)

Temp °C	.0	.1	.2	.3	.4	.5	.6	.7	.8	.9	Derivative
											
	Pa	Pa	Pa	Pa	Pa	Pa	Pa	Pa	Pa	Pa	Pa/deg
											
0	a611.213	615.667	620.150	624.662	629.203	633.774	638.373	643.003	647.662	652.350	44.400
1	657.069	661.819	666.598	671.408	676.249	681.121	686.024	690.958	695.923	700.920	47.340
2	705.949	711.010	716.103	721.228	726.386	731.576	736.799	742.055	747.344	752.667	50.448
3	758.023	763.412	768.836	774.294	779.786	785.312	790.873	796.469	802.100	807.766	53.729
4	813.467	819.204	824.977	830.786	836.631	842.512	848.429	854.384	860.375	866.403	57.192
5	872.469	878.572	884.713	890.892	897.109	903.364	909.658	915.991	922.362	928.773	60.845
6	935.223	941.712	948.241	954.810	961.419	968.069	974.759	981.490	988.262	995.075	64.696
7	1001.93	1008.83	1015.76	1022.74	1029.77	1036.83	1043.94	1051.09	1058.29	1065.52	68.75
8	1072.80	1080.13	1087.50	1094.91	1102.37	1109.87	1117.42	1125.01	1132.65	1140.33	73.03
9	1148.06	1155.84	1163.66	1171.53	1179.45	1187.41	1195.42	1203.48	1211.58	1219.74	77.53
10	1227.94	1236.19	1244.49	1252.84	1261.24	1269.68	1278.18	1286.73	1295.33	1303.97	82.26
11	1312.67	1321.42	1330.22	1339.08	1347.98	1356.94	1365.95	1375.01	1384.12	1393.29	87.24
12	1402.51	1411.79	1421.11	1430.50	1439.93	1449.43	1458.97	1468.58	1478.23	1487.95	92.48
13	1497.72	1507.54	1517.43	1527.36	1537.36	1547.42	1557.53	1567.70	1577.93	1588.21	97.98
14	1598.56	1608.96	1619.43	1629.95	1640.54	1651.18	1661.89	1672.65	1683.48	1694.37	103.75
15	1705.32	1716.33	1727.41	1738.54	1749.75	1761.01	1772.34	1783.73	1795.18	1806.70	109.82
16	1818.29	1829.94	1841.66	1853.44	1865.29	1877.20	1889.18	1901.23	1913.34	1925.53	116.18
17	1937.78	1950.10	1962.48	1974.94	1987.47	2000.06	2012.73	2025.46	2038.27	2051.14	122.85
18	2064.09	2077.11	2090.20	2103.37	2116.61	2129.92	2143.30	2156.75	2170.29	2183.89	129.84
19	2197.57	2211.32	2225.15	2239.06	2253.04	2267.10	2281.23	2295.44	2309.73	2324.10	137.17
20	2338.54	2353.07	2367.67	2382.35	2397.11	2411.95	2426.88	2441.88	2456.94	2472.13	144.84
21	2487.37	2502.70	2518.11	2533.61	2549.18	2564.85	2580.59	2596.42	2612.33	2628.33	152.88
22	2644.42	2660.59	2676.85	2693.19	2709.62	2726.14	2742.75	2759.45	2776.23	2793.10	161.28
23	2810.06	2827.12	2844.26	2861.49	2878.82	2896.23	2913.74	2931.34	2949.04	2966.82	170.07
24	2984.70	3002.68	3020.74	3038.91	3057.17	3075.52	3093.97	3112.52	3131.16	3149.90	179.27
25	3168.74	3187.68	3206.71	3225.85	3245.08	3264.41	3283.85	3303.38	3323.02	3342.76	188.88
26	3362.60	3382.54	3402.59	3422.73	3442.99	3463.34	3483.81	3504.37	3525.05	3545.83	198.91
27	3566.71	3587.71	3608.81	3630.02	3651.33	3672.76	3694.29	3715.94	3737.69	3759.56	209.39
28	3781.54	3803.63	3825.83	3848.14	3870.57	3893.11	3915.77	3938.54	3961.42	3984.42	220.33
29	4007.54	4030.77	4054.12	4077.59	4101.18	4124.88	4148.71	4172.65	4196.71	4220.90	231.75
30	4245.20	4269.63	4294.18	4318.85	4343.64	4368.56	4393.60	4418.77	4444.06	4469.48	243.66
31	4495.02	4520.69	4546.49	4572.42	4598.47	4624.65	4650.96	4677.41	4703.98	4730.68	256.07
32	4757.52	4784.48	4811.58	4838.81	4866.18	4893.68	4921.32	4949.09	4976.99	5005.04	269.01
33	5033.22	5061.53	5089.99	5118.58	5147.32	5176.19	5205.20	5234.36	5263.65	5293.09	282.48
34	5322.67	5352.39	5382.26	5412.27	5442.43	5472.73	5503.18	5533.78	5564.52	5595.41	296.52
35	5626.45	5657.64	5688.97	5720.46	5752.10	5783.89	5815.83	5847.93	5880.17	5912.58	311.13
36	5945.13	5977.84	6010.71	6043.73	6076.91	6110.25	6143.75	6177.40	6211.22	6245.19	326.34
37	6279.33	6313.62	6348.08	6382.70	6417.48	6452.43	6487.54	6522.82	6558.26	6593.87	342.15
38	6629.65	6665.59	6701.71	6737.99	6774.44	6811.06	6847.85	6884.82	6921.95	6959.26	358.60
39	6996.75	7034.40	7072.24	7110.24	7148.43	7186.79	7225.33	7264.04	7302.94	7342.02	375.70
40	7381.27	7420.71	7460.33	7500.13	7540.12	7580.28	7620.64	7661.18	7701.90	7742.81	393.47
41	7783.91	7825.20	7866.67	7908.34	7950.19	7992.24	8034.47	8076.90	8119.53	8162.34	411.92
42	8205.36	8248.56	8291.96	8335.56	8379.36	8423.36	8467.55	8511.94	8556.54	8601.33	431.09
43	8646.33	8691.53	8736.93	8782.54	8828.35	8874.37	8920.59	8967.02	9013.66	9060.51	450.98
44	9107.57	9154.84	9202.32	9250.01	9297.91	9346.03	9394.36	9442.91	9491.67	9540.65	471.63
45	9589.84	9639.25	9688.89	9738.74	9788.81	9839.11	9889.62	9940.36	9991.32	10042.51	493.04
46	10093.92	10145.56	10197.43	10249.52	10301.84	10354.39	10407.18	10460.19	10513.43	10566.91	515.25
47	10620.62	10674.57	10728.75	10783.16	10837.82	10892.71	10947.84	11003.21	11058.82	11114.67	538.28
48	11170.76	11227.10	11283.68	11340.50	11397.57	11454.88	11512.45	11570.26	11628.32	11686.63	562.14
49	11745.19	11804.00	11863.07	11922.38	11981.96	12041.78	12101.87	12162.21	12222.81	12283.66	586.86
50	12344.78	12406.16	12467.79	12529.70	12591.86	12654.29	12716.98	12779.94	12843.17	12906.66	612.47
51	12970.42	13034.46	13098.76	13163.33	13228.18	13293.30	13358.70	13424.37	13490.32	13556.54	638.98
52	13623.04	13689.82	13756.88	13824.23	13891.85	13959.76	14027.95	14096.43	14165.19	14234.24	666.42
53	14303.57	14373.20	14443.11	14513.32	14583.82	14654.61	14725.69	14797.07	14868.74	14940.72	694.81
54	15012.98	15085.55	15158.42	15231.59	15305.06	15378.83	15452.90	15527.28	15601.97	15676.96	724.18
55	15752.26	15827.87	15903.79	15980.02	16056.57	16133.42	16210.59	16288.07	16365.87	16443.99	754.55
56	16522.43	16601.18	16680.26	16759.65	16839.37	16919.41	16999.78	17080.47	17161.49	17242.84	785.95
57	17324.51	17406.52	17488.86	17571.52	17654.53	17737.86	17821.53	17905.54	17989.88	18074.57	818.40
58	18159.59	18244.95	18330.66	18416.71	18503.10	18589.84	18676.92	18764.35	18852.13	18940.26	851.93
59	19028.74	19117.58	19206.76	19296.30	19386.20	19476.45	19567.06	19658.03	19749.35	19841.04	886.56
60	19933.09	20025.51	20118.29	20211.43	20304.95	20398.82	20493.07	20587.69	20682.68	20778.05	922.33
61	20873.78	20969.90	21066.39	21163.25	21260.50	21358.12	21456.13	21554.51	21653.28	21752.44	959.25
62	21851.98	21951.91	22052.23	22152.93	22254.03	22355.52	22457.40	22559.68	22662.35	22765.42	997.35
63	22868.89	22972.75	23077.02	23181.69	23286.76	23392.23	23498.12	23604.40	23711.10	23818.20	1036.66
64	23925.72	24033.65	24141.99	24250.74	24359.91	24469.50	24579.51	24689.93	24800.78	24912.04	1077.21
65	25023.74	25135.85	25248.39	25361.36	25474.76	25588.58	25702.84	25817.53	25932.66	26048.22	1119.03
66	26164.21	26280.64	26397.52	26514.83	26632.58	26750.78	26869.42	26988.51	27108.04	27228.02	1162.14
67	27348.46	27469.34	27590.68	27712.46	27834.71	27957.41	28080.57	28204.19	28328.26	28452.80	1206.57
68	28577.81	28703.28	28829.21	28955.61	29082.48	29209.82	29337.64	29465.92	29594.68	29723.92	1252.36
69	29853.63	29983.82	30114.49	30245.65	30377.28	30509.40	30642.01	30775.10	30908.68	31042.75	1299.52
70	31177.32	31312.37	31447.92	31583.97	31720.51	31857.55	31995.09	32133.14	32271.68	32410.73	1348.09
71	32550.29	32690.35	32830.93	32972.01	33113.61	33255.71	33398.34	33541.48	33685.13	33829.31	1398.10
72	33974.01	34119.23	34264.97	34411.24	34558.03	34705.36	34853.21	35001.59	35150.51	35299.96	1449.58
73	35449.95	35600.47	35751.54	35903.14	36055.29	36207.98	36361.21	36514.99	36669.32	36824.20	1502.56
74	36979.63	37135.61	37292.15	37449.24	37606.89	37765.10	37923.87	38083.21	38243.10	38403.56	1557.06
75	38564.59	38726.19	38888.36	39051.10	39214.41	39378.30	39542.76	39707.80	39873.42	40039.63	1613.13
76	40206.41	40373.78	40541.74	40710.28	40879.42	41049.14	41219.46	41390.37	41561.88	41733.99	1670.78
77	41906.69	42080.00	42253.91	42428.42	42603.54	42779.27	42955.61	43132.55	43310.11	43488.29	1730.06
78	43667.08	43846.48	44026.51	44207.16	44388.43	44570.33	44752.85	44936.00	45119.77	45304.18	1790.99
79	45489.23	45674.91	45861.22	46048.17	46235.76	46424.00	46612.87	46802.39	46992.56	47183.38	1853.60
80	47374.85	47566.97	47759.74	47953.17	48147.25	48342.00	48537.40	48733.47	48930.20	49127.60	1917.93
81	49325.67	49524.40	49723.81	49923.89	50124.64	50326.08	50528.19	50730.98	50934.45	51138.61	1984.01
82	51343.45	51548.98	51755.20	51962.11	52169.72	52378.01	52587.01	52796.70	53007.10	53218.20	2051.86
83	53430.00	53642.50	53855.72	54069.64	54284.28	54499.63	54715.69	54932.47	55149.97	55368.19	2121.53
84	55587.13	55806.80	56027.20	56248.32	56470.17	56692.76	56916.08	57140.13	57364.92	57590.45	2193.05
85	57816.73	58043.74	58271.51	58500.02	58729.27	58959.28	59190.05	59421.57	59653.84	59886.87	2266.45
86	60120.67	60355.23	60590.55	60826.64	61063.50	61301.12	61539.52	61778.70	62018.65	62259.38	2341.76
87	62500.89	62743.18	62986.26	63230.12	63474.78	63720.22	63966.45	64213.48	64461.31	64709.93	2419.01
88	64959.35	65209.58	65460.61	65712.45	65965.09	66218.55	66472.82	66727.90	66983.80	67240.52	2498.25
89	67498.06	67756.42	68015.60	68275.62	68536.46	68798.13	69060.64	69323.98	69588.15	69853.17	2579.50
90	70119.03	70385.73	70653.28	70921.67	71190.91	71461.01	71731.96	72003.76	72276.42	72549.95	2662.79
91	72824.33	73099.58	73375.70	73652.68	73930.54	74209.27	74488.87	74769.35	75050.71	75332.95	2748.17
92	75616.07	75900.08	76184.98	76470.77	76757.44	77045.02	77333.49	77622.86	77913.13	78204.30	2835.66
93	78496.38	78789.36	79083.26	79378.06	79673.78	79970.42	80267.97	80566.45	80865.85	81166.17	2925.31
94	81467.42	81769.60	82072.71	82376.75	82681.73	82987.65	83294.51	83602.31	83911.06	84220.75	3017.14
95	84531.40	84842.99	85155.54	85469.05	85783.51	86098.94	86415.33	86732.68	87051.00	87370.29	3111.19
96	87690.56	88011.80	88334.01	88657.20	88981.38	89306.54	89632.68	89959.82	90287.94	90617.06	3207.50
97	90947.17	91278.28	91610.39	91943.50	92277.62	92612.74	92948.87	93286.02	93624.18	93963.35	3306.10
98	94303.54	94644.76	94986.99	95330.26	95674.55	96019.87	96366.23	96713.62	97062.05	97411.51	3407.03
99	97762.02	98113.58	98466.18	98819.83	99174.54	99530.30	99887.11	100244.99	100603.93	100963.93	3510.33
100	101324.99										

a Metastable state

## Footnotes

1 Figures in brackets indicate the literature references at the end of this paper.

2 1 Pa = 1N/m^2^=10^−5^ bar=10^−2^ mb=7.50062×10^−3^ mm Hg.

3 1 international joule=1.000165 (absolute) joule.

4 According to Eisenberg and Kauzman [9], who quote Shatenshtein et al. [10], the isotopic content of naturally occuring water depends on its origin. Within the limits of variation normally encountered the isotopes are H_2_^16^O, H_2_^17^O, H_2_^18^O, and HDO and their abundances are 99.73, 0.04, 0.20, and 0.03 percent, respectively. Combining these abundances with the relative atomic masses of the appropriate nuclides, recommended by the Commission on Atomic Weights [11], yielded the value 18.01528 grams for the molecular weight of naturally occurring water.

5 In 1972 Ambrose and Lawrenson [20] published an empirical equation in the form of a Chebyshev polynomial for which the constants were obtained by fitting the data of Stimson [4] (25 to 100°C), Beattie and Blaisdell [21] (96 to 103 °C) and Osborne, Stimson, Fiock, and Ginnings [22] (100°C to critical temperature). The other formulations are discussed in ref [1].
